# Space–time distribution of intestinal infectious diseases and their association with socioeconomic variables in Ecuador

**DOI:** 10.3389/fpubh.2024.1412362

**Published:** 2024-07-10

**Authors:** Karina Lalangui, Max Cotera-Mantilla, Marco Sánchez-Murillo, Alex Carrera-Alvarez, Mónica Duque-Cuasapaz, Emmanuelle Quentin

**Affiliations:** ^1^Centro de Investigación EpiSIG, Instituto Nacional de Investigación en Salud Pública, Quito, Ecuador; ^2^Centro de Investigación en Salud Pública y Epidemiología Clínica (CISPEC), Facultad Ciencias de la Salud Eugenio Espejo, Universidad UTE, Quito, Ecuador

**Keywords:** intestinal infectious diseases, spatial clusters, spatiotemporal analysis, Ecuador, socioeconomic variables

## Abstract

**Background:**

Intestinal infectious diseases are a global concern in terms of morbidity, and they are closely linked to socioeconomic variables such as quality of life, weather and access to healthcare services. Despite progress in spatial analysis tools and geographic information systems in epidemiology, studies in Ecuador that evaluate temporal trends, specific geographic groups, and their correlation with socioeconomic variables are lacking. The absence of such information makes it challenging to formulate public health policies. This study sought to identify the spatial and temporal patterns of these diseases in Ecuador, along with their correlation with socioeconomic variables.

**Methods:**

In Ecuador, the study was carried out in a continental territory, focusing on data related to intestinal infectious diseases collected from the National Institute of Statistics and Census (Instituto Nacional de Estadística y Censos) during the period from 2014 to 2019. This study involved spatial and temporal analyses using tools such as the global Moran’s index and Local Indicators of Spatial Association to identify spatial clustering patterns and autocorrelation. Additionally, correlations between morbidity rates and socioeconomic variables were examined.

**Results:**

During the investigated period, Ecuador registered 209,668 cases of these diseases. Notable variations in case numbers were identified, with a 9.2% increase in 2019 compared to the previous year. The most impacted group was children under 5 years old, and the highest rates were centered in the southern and southwestern regions of the country, with Limón Indanza and Chunchi being the cantons with the highest rates, notably showing a significant increase in Limón Indanza. Additionally, there were significant correlations between morbidity rates and socioeconomic variables, school dropout rates, low birth weight, and access to water services.

**Conclusion:**

This study emphasizes the importance of considering socioeconomic variables when addressing these diseases in Ecuador. Understanding these correlations and geospatial trends can guide the development of health policies and specific intervention programs to reduce the incidence in identified high-risk areas. More specific research is needed to understand the underlying causes of variability in morbidity and develop effective prevention strategies.

## 1 Introduction

Intestinal infectious diseases persist as major contributors to morbidity on a global scale (1–5). According to the World Health Organization (WHO), access to clean water, adequate sanitation services, and proper hygiene in households could have prevented the loss of at least 1.4 million lives and 74 million disability-adjusted life years (DALYs) (6). These diseases represent a significant burden, particularly in low- and middle-income countries, where poor living conditions and limited access to health services exacerbate the problem (6, 7). Recent studies have highlighted the importance of socioeconomic factors such as poverty and quality of life (8–10) in the prevalence of these diseases. In addition, nutritional factors (10), climatic factors (5, 11, 12) and the availability of health services (13) also play a crucial role in their incidence.

The field of epidemiology has undergone remarkable progress with the implementation of spatial analysis tools and geographic information systems. These tools enable the analysis of the temporal and spatial distribution of intestinal infectious diseases, facilitating the identification of areas with higher risk and associated factors (14–16), thereby greatly enhancing decision-making efficiency in public health. For example, in Haiti, a study used geographic information systems (GIS) to analyze spatial determinants and identify priority locations for cholera prevention interventions (17). In Peru, several studies have used spatial analysis to assess socioeconomic inequalities and analyze the spatial distribution of childhood diarrhea, providing important suggestions for improving the planning and effectiveness of public health interventions (18, 19).

In Ecuador, although intestinal infections have a significant impact on children under 11 years of age (20), especially in vulnerable communities (21), there are few studies that evaluate temporal trends and specific geographic groups related to intestinal diseases, and that establish a strong association between these diseases and social factors (22). The majority of studies are case–control studies and have concentrated on analyzing specific regions (21, 23, 24). This information gap poses a significant challenge in decision-making and public policy formulation in the country. Therefore, this study aimed to identify spatial and temporal patterns in the distribution of intestinal infectious diseases in Ecuador and explore potential global and local autocorrelation with socioeconomic variables. This could facilitate the prioritization of areas requiring specific preventive measures.

## 2 Methods

### 2.1 Study area

**Figure 1:**
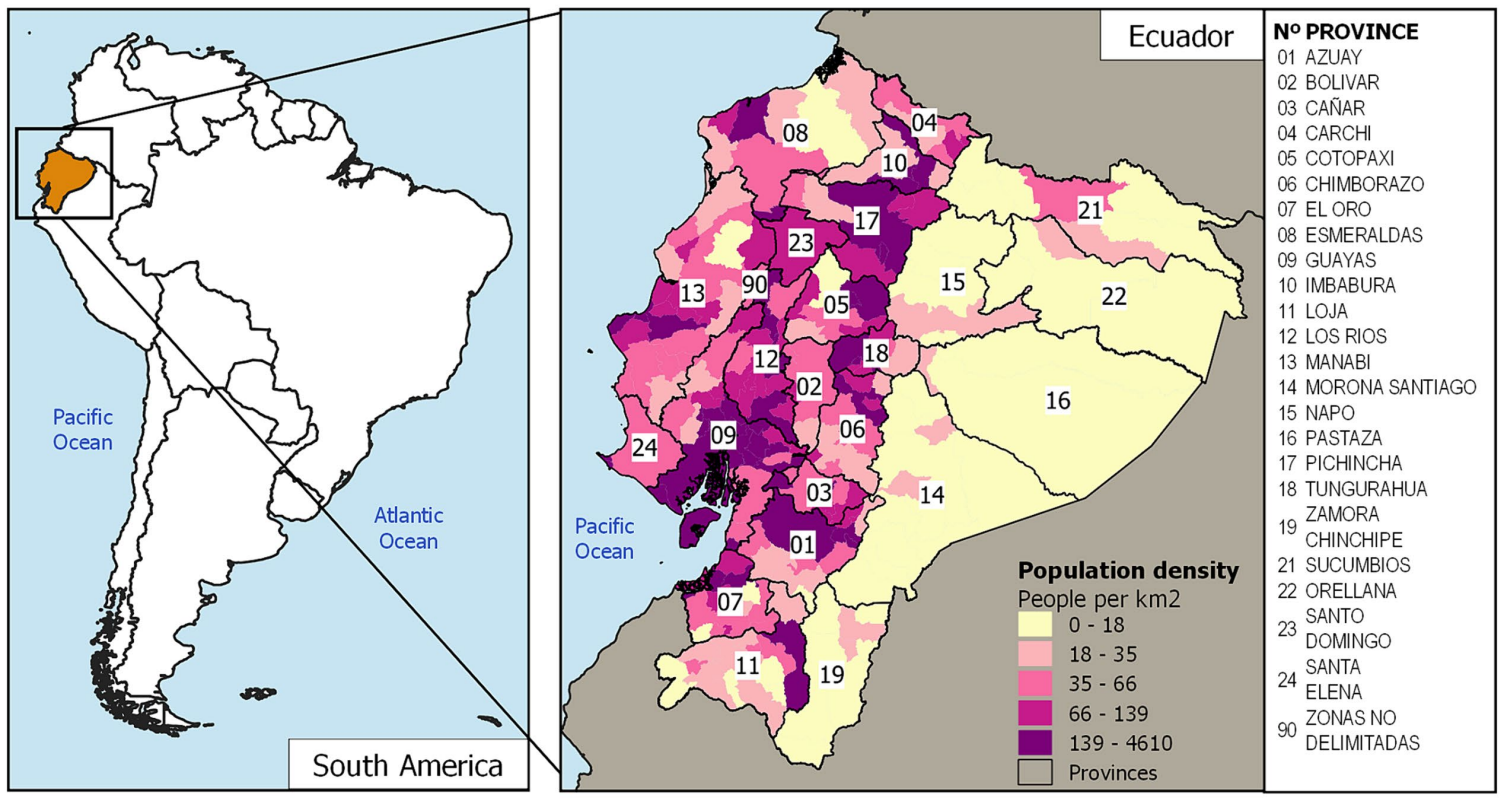
Map of the study area. Cantons of Ecuador with their population density. Data are from the INEC database.

Located in South America, Ecuador has a total area of 256,370 square kilometers and a population of 17,268,012 as of 2019 (25). The country is divided into 24 provinces, 221 cantons (equivalent to municipalities), and 3 zones without political delimitation. For the analysis, the information is restricted to the administrative-political division of 2012 (last updated shapefile) (26), focusing on continental Ecuador with 23 provinces (excluding code 20 assigned to Galápagos), which accounts for 218 cantons, plus the 3 undelimited zones. (Figure 1). Methodological considerations have led to the exclusion of the province of Galapagos from this analysis. As an archipelago comprising distinct islands, it does not meet the neighborhood criteria necessary for the spatial analysis, which is based on geographic proximity for the identification of clusters. Furthermore, the number of cases of intestinal infectious diseases is low, which does not provide significant results for this study.

### 2.2 Data

Intestinal infectious diseases were categorized based on the International Classification of Diseases (ICD-10). These diseases include intestinal infectious diseases (A00-A09), acute hepatitis (B15), schistosomiasis (B65), hookworm disease (B76), ascariasis (B77), and trichuriasis (B79) (27). All the codes for the selected diseases were verified to match those in the databases utilized for this study. Information regarding these diseases, categorized by canton of residence, was extracted from the hospital discharge records maintained by the National Institute of Statistics and Census (Instituto Nacional de Estadística y Censos, INEC) for the years 2014 to 2019 (28). The hospital discharge dataset includes data reported by health facilities offering inpatient services, encompassing the comprehensive public health network and the complementary network. Data from 2020 to 2021 were not considered for the analysis. This exclusion was a result of measures taken to control the COVID-19 pandemic, including mobility restrictions, lockdowns, and quarantine, which led to limitations affecting regular access to public and private health facilities (29). Population projection data from the INEC (25) were used for rate calculations. The socioeconomic variables data on inadequate sanitation, access to water, and waste collection services were obtained from the 2010 Population and Housing Census of the INEC (Censo de población y vivienda del INEC 2010) (30). Infant mortality data were collected from the 2019 General Mortality dataset provided by the INEC (31). Additionally, the dropout rate and the percentage of children with low birth weight, which are indicators of educational level (32), were calculated using the 2019 educational information databases from the Ministry of Education of Ecuador (Ministerio de Educación de Ecuador) (33) and the statistical records of live births and fetal deaths from INEC 2019 (34), respectively. All the data are publicly available and are published on institutional websites.

### 2.3 Temporal analysis

**Figure 2:**
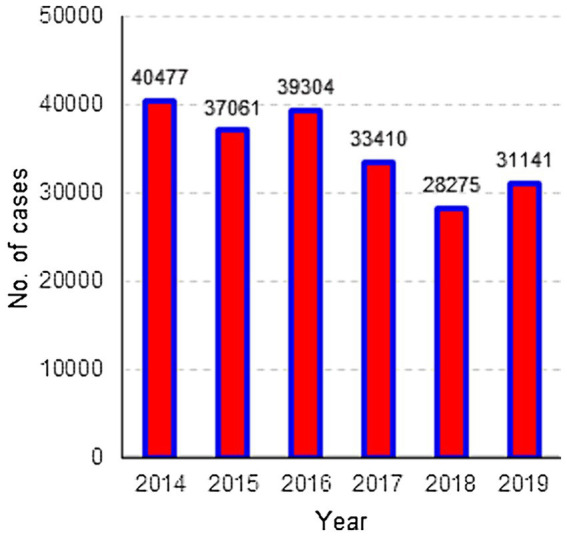
Distribution of cases of intestinal infectious diseases, Ecuador 2014–2019.

The first step involved creating maps illustrating the spatial distribution of the rates of intestinal infectious diseases from 2014 to 2019. These rates were presented on choropleth maps and categorized into five groups (Figure 2). Additionally, the number of years that a specific canton had higher rates of intestinal infectious diseases than the national average was determined by calculating, for each year, a rate of the national average and assigning a value of one when the observed rate was higher than the national average rate and 0 when the observed rate was lower than the national average rate.

The analysis of temporal trends is performed using the median trend method (Theil-Sen), a robust nonparametric operator widely recommended for evaluating the rate of change in short and noisy series (35). This method is capable of handling up to 29% of missing data and still produces reliable results (36). This method is particularly relevant in the context of health data, where series may be incomplete or inconsistent. Furthermore, calculating the median slope between pairs of points provides a robust and efficient estimate of the central tendency, as it is less susceptible to outliers than traditional parametric methods. The slope between each pair combination is calculated, and the median value is identified (37). Only areas with a significance level of *p* < 0.05 were considered. It should be noted, however, that by establishing a significance threshold, it becomes possible to exclude areas that may have a certain trend but are not statistically significant at the level being considered. The temporal analysis was performed using TerrSet software, and maps were generated using QGIS 3.4.8.

### 2.4 Spatial analysis

To verify whether spatial distribution depends on space, a spatial autocorrelation analysis is conducted using the univariate Moran’s index (UMI) (38, 39). The UMI measures the degree of spatial dependence or average spatial autocorrelation. Spatial autocorrelation is indicated by the z score and *p* value, where values close to +1 indicate perfect positive autocorrelation, values close to −1 indicate perfect negative autocorrelation, and a value of 0 indicates the presence of completely random patterns in spatial distribution (40, 41). For this analysis, the queen contiguity criterion was used, with a significance level of 5%.

Spatial autocorrelation at the local level was evaluated using Local Indicators of Spatial Association (LISA). This method indicates the locations of clusters, thereby indicating the presence of hotspots or outliers. By means of this analysis, four grouping categories based on the type of spatial association can be identified: Q1 (high-high or hotspot) corresponds to values above the average, which, in turn, have neighbors with high values. The opposite situation is shown in Q2 (low-low or cold spots). Both groupings detect clusters with similar values to their neighbors. In contrast, spatial outliers indicate cantons with discordant values among neighbors and no spatial association; these are Q3 (low-high), values below the average with neighbors having high values. On the other hand, the opposite scenario is found for Q4 (high-low) (42). The spatial analysis was applied to the rates of the last year of study.

### 2.5 Autocorrelation analysis with socioeconomic variables

The analysis started by assessing spatial dependence using UMI statistics. Global and local bivariate Moran index tests were then performed. Bivariate spatial correlation calculations were applied using municipal indicators as independent variables and the 2019 morbidity rate as the dependent outcome. Bivariate Moran’s analysis (BMI) is a technique that helps to determine if there is a spatial relationship between the values of two observed variables in different regions. More precisely, it assists in determining whether the value of an attribute in a specific region is spatially correlated with the values of another variable in adjacent regions. In other words, BMI helps measure the degree of spatial linear correlation, whether positive or negative, between the value of one variable and the average of another variable in adjacent locations (43). The results were considered statistically significant if a *p*- value <0.05 was obtained. Spatial analysis was performed using Geoda 1.20, and maps were generated using QGIS 3.4.8.

## 3 Results

### 3.1 Descriptive analysis

In total, 209,668 cases of intestinal infectious diseases were recorded between 2014 and 2019. The highest number of cases, 40,477, was recorded in 2014. The lowest number of cases, 28,275, occurred in 2018, and for the year 2019, the number of cases increased by 9.2% compared to that in the previous year (Figure 2).

Regarding the epidemiological characteristics of the cases recorded from 2014 to 2019, Table 1 shows the percentage distribution of cases by sex, age group, and ethnicity. For the gender variable, the proportion of the female population is greater than that of the male population; however, there is no significant difference in the proportion of men and women. In the case of age groups, the highest proportion of cases occurred in the 0–5 age group, with percentages ranging between 33.81 and 39.64%. The 6–14 age group also had a significant proportion of cases, with percentages remaining relatively stable at approximately 22%. Other age groups show lower percentages of cases, with small variations over the years. The most common ethnic group was the mestizo group, with percentages ranging from 81.31 to 89.43% over the years.

**Table 1:** Epidemiological characteristics of cases with intestinal infectious diseases in Ecuador from 2014 to 2019.

	**No. of cases 2014**	**%**	**No. of cases 2015**	**%**	**No. of cases 2016**	**%**	**No. of cases 2017**	**%**	**No. of cases 2018**	**%**	**No. of cases 2019**	**%**
Sex												
Male	19,932	49.24	18,332	49.46	19,400	49.36	16,461	49.27	13,625	48.19	15,209	48.84
Female	20,545	50.76	18,729	50.54	19,904	50.64	16,949	50.73	14,650	51.81	15,932	51.16
Age group												
0–5	14,604	36.08	14,044	37.89	15,579	39.64	12,189	36.48	9,559	33.81	11,531	37.03
6–14	8,817	21.78	8,324	22.46	8,899	22.64	7,562	22.63	6,490	22.95	7,201	23.12
15–24	3,283	8.11	2,845	7.68	2,767	7.04	2,545	7.62	2,225	7.87	2,234	7.17
25–34	3,479	8.60	2,922	7.88	2,816	7.16	2,587	7.74	2,341	8.28	2,381	7.65
35–44	2,536	6.27	2,211	5.97	2,267	5.77	1971	5.90	1818	6.43	1833	5.89
45–54	2,173	5.37	1869	5.04	1818	4.63	1,667	4.99	1,477	5.22	1,494	4.80
55–64	1924	4.75	1,661	4.48	1,684	4.28	1,606	4.81	1,442	5.10	1,467	4.71
65+	3,661	9.04	3,185	8.59	3,474	8.84	3,283	9.83	2,923	10.34	3,000	9.63
Ethnicity												
Indigenous	1,373	3.39	1,377	3.72	1,265	3.22	1,344	4.02	1,114	3.94	1,203	3.86
Afro-Ecuadorian	184	0.45	166	0.45	209	0.53	92	0.28	142	0.50	109	0.35
Black	76	0.19	58	0.16	60	0.15	85	0.25	81	0.29	46	0.15
Mulato	55	0.14	56	0.15	184	0.47	45	0.13	51	0.18	62	0.20
Montubio	57	0.14	50	0.13	84	0.21	198	0.59	194	0.69	86	0.28
Mestizo	32,913	81.31	30,675	82.77	35,148	89.43	29,607	88.62	24,344	86.10	26,724	85.82
White	498	1.23	345	0.93	437	1.11	339	1.01	274	0.97	240	0.77
Other	758	1.87	984	2.66	835	2.12	573	1.72	695	2.46	619	1.99
Unknown	4,563	11.27	3,350	9.04	1,082	2.75	1,127	3.37	1,380	4.88	2052	6.59

### 3.2 Temporal analysis

**Figure 3:**
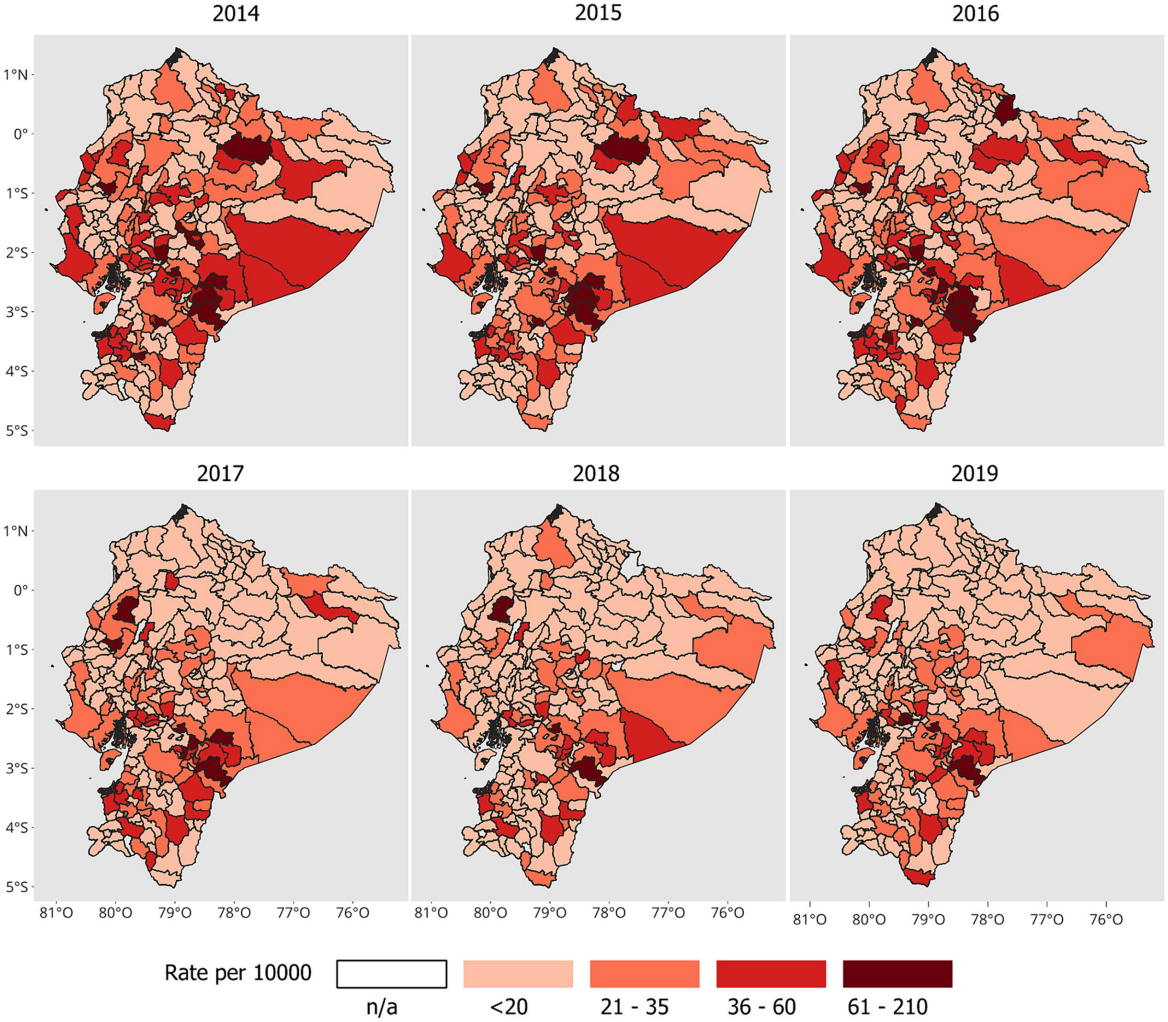
Spatial distribution of morbidity rates of intestinal infectious diseases, 2014–2019.

The morbidity rates of intestinal infectious diseases also varied over time, as illustrated in Figure 3. Initially, temporal similarities in spatial patterns were observed between 2014 and 2016, with certain cantons maintaining comparable rates during this period (refer to Table 1 for specific details). From 2017 to 2019, a decrease in spatial patterns was recorded, especially in the northern and northeastern regions of Ecuador. For the year 2019, the cantons that had the most cases in the range of 61 to 210 per 10,000 inhabitants were Limón Indanza (Morona Santiago), Coronel Marcelino Maridueña (Guayas), Naranjito (Guayas), and Chunchi (Chimborazo).

**Figure 4:**
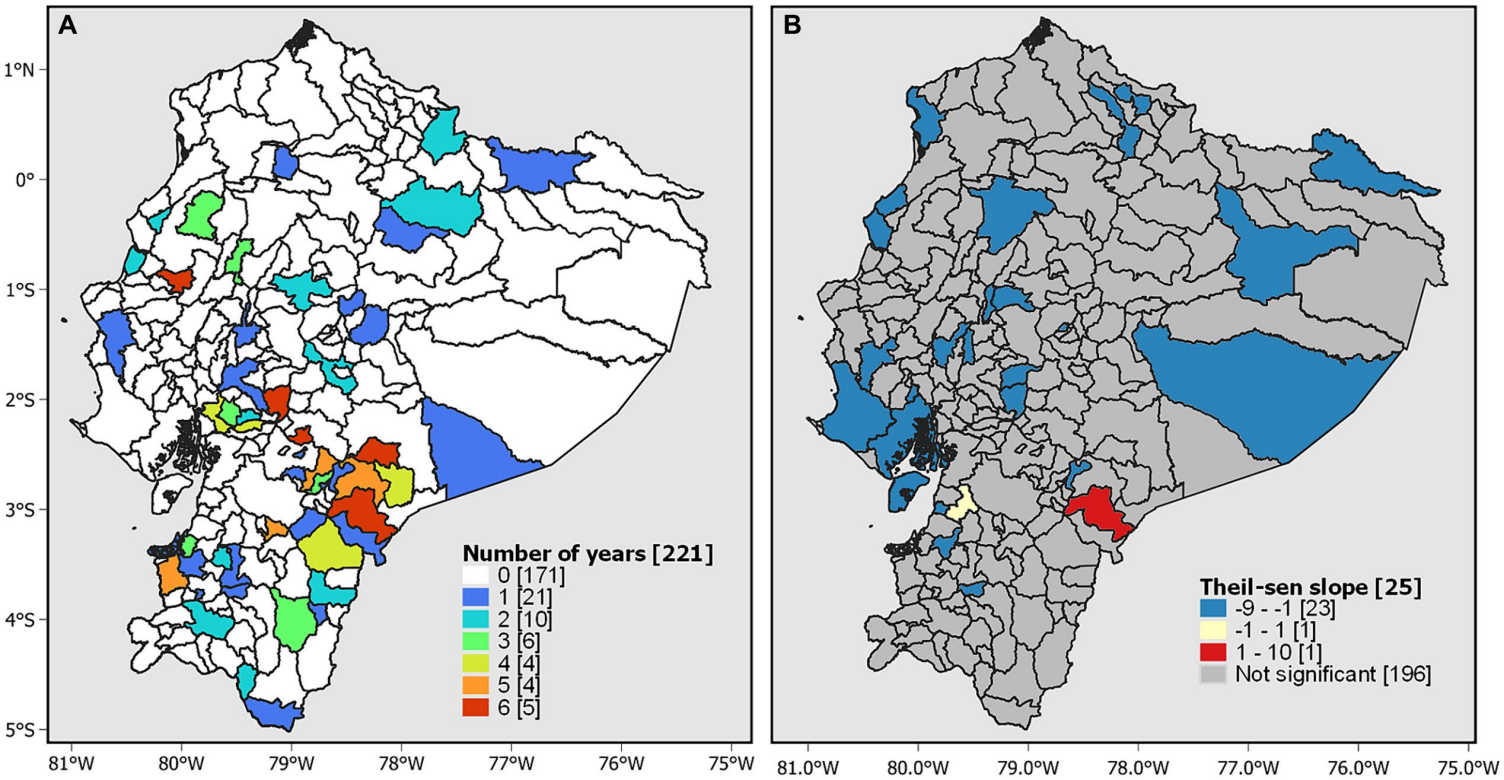
Temporal analysis. **(A)** Morbidity rate higher than expected and **(B)** trends in morbidity rates based on the Theil–Sen estimator.

Cantons such as Sucúa and Limón Indanza (Morona Santiago), Bolívar (Manabí), Chunchi (Chimborazo), and Chillanes (Bolívar) had higher-than-expected rates over the 6 years of the study (Figure 4A). The trend of the Theil-Sen estimator was significant (*p* < 0.05) in 25 cantons, and Limón Indanza (Morona Santiago) was the only one showing an increasing trend in the morbidity rates of intestinal infectious diseases (Figure 4B). The other cantons with higher-than-expected rates did not demonstrate significant differences according to this method.

### 3.3 Spatial analysis

**Figure 5:**
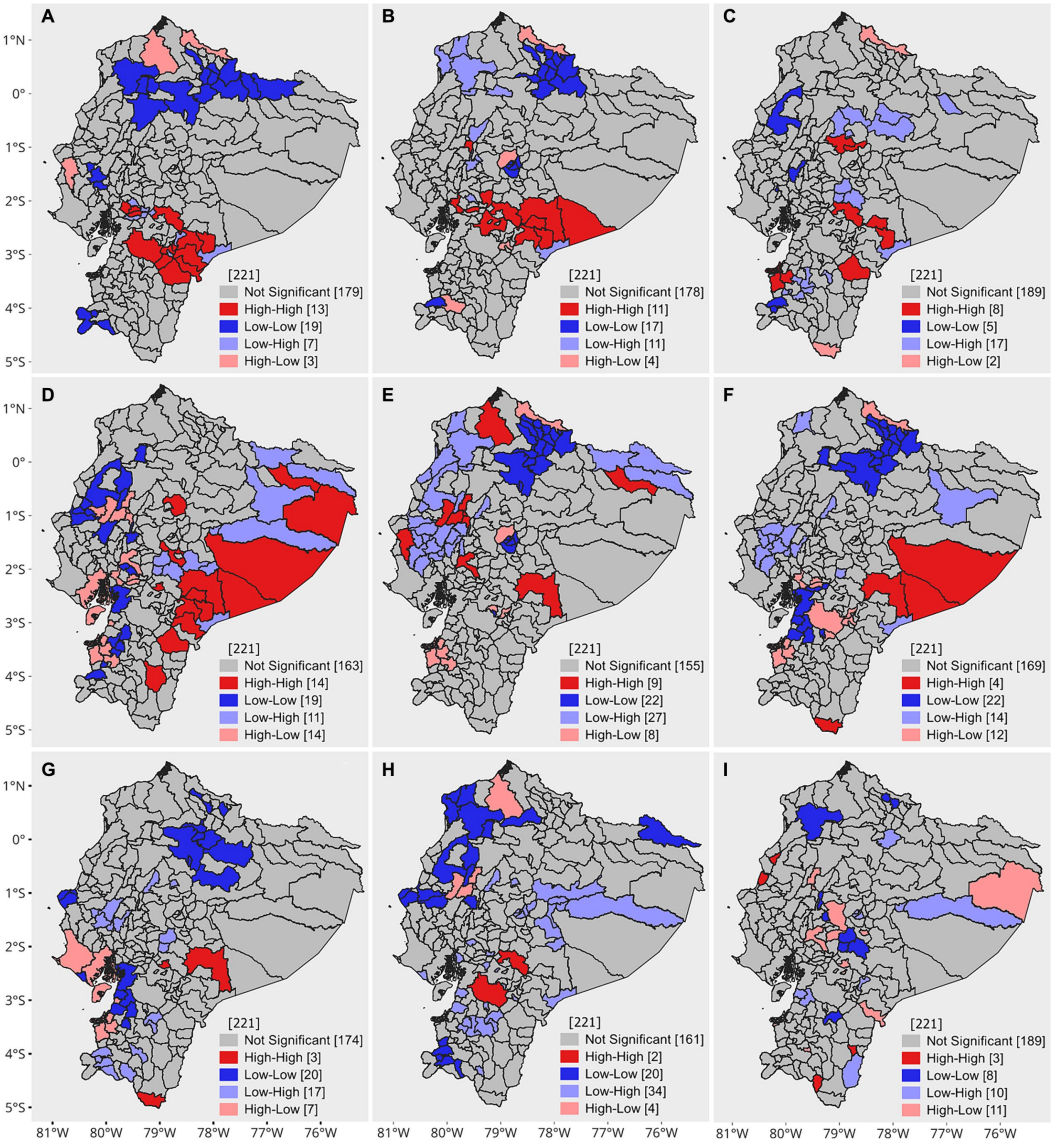
Univariate and bivariate LISA clustering. **(A)** Univariate LISA 2019, **(B)** percentage of school dropouts, **(C)** infant mortality rate per 1,000 live births, **(D)** percentage of children with low birth weight, **(E)** percentage of households without access to the public water network, **(F)** percentage of households without access to sewage, **(G)** percentage of households without access to garbage collection, **(H)** continuity of water service in urban areas (hours/day), and i. continuity of water service in rural areas (hours/day).

The global Moran’s index for 2019 shows a moderate positive spatial autocorrelation (0.22), and the *p* value (0.00005) and z value (5.36) indicate that the spatial autocorrelation is statistically significant. This suggests that the morbidity rates of diseases tend to cluster spatially in a similar way in Ecuador. The LISA spatial autocorrelation analysis revealed a high-high (Q1) dependency pattern in 14 cantons located in the central-southern part of Ecuador. Alternatively, 19 cantons exhibited a low-low (Q2) pattern and were located in the northern part of Ecuador (Figure 5A).

With respect to bivariate autocorrelation, the percentage of school dropouts, the percentage of children with low birth weight, and the continuity of service in urban areas were significantly correlated with the morbidity rates of intestinal infectious diseases (*p* < 0.05) (Table 2); however, there was no strong spatial correlation. In contrast, the bivariate LISA results showed local patterns of spatial correlation. For the percentage of school dropouts, infant mortality rate per 1,000 live births, households without access to the public water network, households without access to sewage, and households without access to garbage collection, high-high values indicate cantons with a high morbidity rate of intestinal infectious diseases surrounded by cantons with high percentages of each of the mentioned variables. The high-high clusters are predominantly grouped in the Amazon and the central Sierra, except for the variable of households without access to the public water network, where clusters are observed in some coastal provinces. Regarding the continuity of water services in urban and rural areas, the most relevant clusters have a high-low configuration. This is because high morbidity rates are surrounded by cantons where the availability of water service hours is low (Figure 5).

**Table 2:** Global bivariate Moran’s index for each indicator.

Indicators	I Moran	*p*	*z*
Student dropout percentage	0.116	0.00066	3.6343
Infant mortality rate (< 5 years)	0.027	0.1754	0.8539
Percentage of low-birth-weight children	0.086	0.008	2.6433
Homes without access to public water network	−0.027	0.19099	−0.8738
Homes without access to sewage	0.004	0.4547	0.1066
Homes without access to garbage collection	0.023	0.2358	0.7237
Continuity of water service in urban areas (hours/day)	0.128	0.00002	3.9899
Continuity of water service in rural areas (hours/day)	0.029	0.1824	0.9124

## 4 Discussion

Epidemiological studies on infectious diarrhoeal and intestinal diseases play a crucial role in public health for preventing such infections. Identifying areas with higher or lower incidence, for example, through choropleth maps, allows for targeted intervention solutions in the most affected zones. However, a more in-depth analysis, using spatio-temporal analysis methods alongside the assessment of contributing factors to the manifestation of these pathologies, provides a more precise graphical representation of locations with a greater predisposition to the occurrence of these diseases (3, 8, 9).

This study focused on analyzing the morbidity of hospitalizations due to intestinal infectious diseases in Ecuador during the period of 2014–2019. Through space–time analysis, we identified epidemiological characteristics, temporal trends, spatial clusters, and spatial autocorrelations with socioeconomic variables.

The data observed in this study have significant implications for public health strategies. The identification of spatial clusters of high incidence of intestinal infectious diseases allows for the targeting of public health interventions to specific areas with greater needs. For instance, the implementation of health education and sanitation infrastructure improvement programs in cantons such as Sucúa, Limón Indanza, Bolívar., Chunchi and Chillanes could be an effective strategy. Systematic review studies have evaluated the effectiveness of education and sanitation interventions to prevent diarrheal diseases (44, 45). Consequently, integrating spatio-temporal analysis with socioeconomic data can enhance the scientific and rational nature of health resource planning (46).

When analyzing temporal trends, we observed significant variation in the number of cases over the years. The highest number of cases was recorded in 2014, while the lowest number of cases was recorded in 2018. However, it is important to note that 2019 experienced a 9.2% increase compared to the previous year. This variability could be related to various factors, such as changes in case reporting, seasonal variations (47, 48), or the effectiveness of preventive measures (49).

When examining the epidemiological characteristics, we noticed that the age group of 0–5 years was the most affected, with percentages ranging from 33.81 to 39.64%. Previous research has indicated that children under 5 years old are at high risk for these diseases (9, 24, 50).

The findings indicate that identifying specific demographic regions and vulnerable groups at higher risk is crucial for implementing effective preventive measures. Promoting early and exclusive breastfeeding is a key strategy for preventing diarrhea and hospital admissions among infants. A 2016 review on trends in breastfeeding throughout the world suggests that this practice prevents half of diarrhea incidents and 72% of hospitalizations (51). Furthermore, maintaining proper hand hygiene with soap and water is an effective strategy for reducing the transmission of intestinal pathogens (44).

Rotavirus represents the main cause of severe diarrhea in the infant population. Annually, this infection causes the death of more than half a million children under 5 years old and leads to millions of more hospitalizations (52). In Ecuador, vaccination coverage, including rotavirus, reached 100% until 2009 and then declined considerably until 2016 (53). Starting in 2016, the decreasing trend reversed, and the coverage increased; however, when disaggregated to smaller geographical levels, significant heterogeneity was evident, with coverages ranging from 60 to 80% (54, 55). This decline in vaccination could contribute to the increase in cases of intestinal infectious diseases, especially in children, emphasizing the need to address not only the effectiveness of vaccination but also the obstacles that may affect its implementation and access at the local level.

The use of geoprocessing methods allowed the detection of spatial patterns that describe how hospital cases of intestinal infectious diseases are distributed during the period of 2014–2019. In 2019, the highest rates of intestinal infectious diseases were concentrated in southern and southwestern Ecuador, mainly in rural areas of the provinces of Morona Santiago, Guayas, and Chimborazo. Compared with the other cantons, Limón Indanza and Chunchi were the cantons with the highest rates, which were higher than expected in the 6 years of study. Interestingly, only Limón Indanza showed a significant increasing trend in morbidity rates (*p* < 0.05), suggesting a consistent increase in morbidity in this canton, possibly related to sewage contamination and extensive livestock farming in water intakes intended for human consumption (56). A study in Ecuador revealed that Limón Indanza is the second most affected canton in terms of the average age of death from waterborne diseases, with 844.98 cases per 100,000 inhabitants (22).

The analysis also revealed significant global correlations between the morbidity rates of intestinal infectious diseases and various socioeconomic and health indicators. The percentage of student dropouts, the percentage of children with low birth weight, and the continuity of water service in urban areas showed significant correlations (*p* < 0.05), although they were not particularly strong. It is important to note that the percentage of student dropout and the prevalence of low birth weight presented high-high clusters that were mainly grouped in rural areas of the Amazon and the central highlands. These areas, which are generally populated by indigenous communities with lower education levels and less access to basic services, are at greater risk of unhygienic practices, such as lack of handwashing after using the toilet or before meals, open-to-river defecation, and poor waste management. These factors can contribute to the increased incidence of intestinal infectious diseases in these communities (57).

Some practical measures in sectors where high-high clusters exist could focus on educating mothers about the importance of proper nutrition to reduce the risk of low birth weight infants. A study in Bangladesh showed that community education in resource-poor settings on balanced nutrition during pregnancy reduces the risk of low birth weight infants (58). In addition, implementing school retention programs can help reduce school dropout. Combining these programs with health education can improve the capacity of communities to adopt appropriate health practices.

Health education as part of health promotion plays a crucial role in disease transmission, representing between 32 and 39%, according to a study by the Department of Water Resources Engineering at the University of Dar es Salaam (1).

The variable related to access to water services did not have a significant global correlation with morbidity rates of intestinal infectious diseases. A study conducted in Ecuador on the coping strategies of households associated with water supply and diarrhea revealed that access to domestic water connections was not significantly associated with diarrhea. The lack of reliability in tap water leads households to use strategies to cope with unsafe water (59). The high-high clusters for this variable are present in certain cantons on the coast, where it is also interesting to note that there is an extensive area of low-high clusters, which could indicate that home water treatment strategies might contribute to the low incidence of diarrhoeal diseases.

This study may have limitations related to the quality of hospital discharge statistics and the updating of some socioeconomic data. This could impact the accuracy of the spatial correlation analysis. Furthermore, the time lag between the databases used for socioeconomic variables and morbidity data could affect the accuracy of the correlation analysis, however, these databases are considered among the most reliable for a study of this nature. Despite these limitations, the results allow for relative comparisons in space and in time, which minimizes the impact of uncertainty in absolute values.

Another possible limitation is the lack of causal analysis, highlighting the need for additional research to better understand the factors behind the observed trends. At an ecological level (not individual), correlation is not sufficient to imply causality, and the bivariate method does not take into account the interrelation between determinants. It should be noted that the municipal indicators are only available for the census years of 2010 and 2022. It may be the case that disease rates are more stable over time; however, it is clear that a method must be developed or applied to estimate the values of these indicators between census dates.

For future research, the most recent socio-economic data from the 2022 census can be utilized, and the analysis can be expanded to include disease data from the same period. It would also be beneficial to incorporate environmental variables, particularly in the context of climate change, as these factors can significantly impact the incidence of intestinal diseases. The use of advanced methods to address causality and understand the interdependence of variables would provide a more comprehensive understanding of the dynamics underlying the morbidity of these diseases. This will enable the development of more effective interventions, tailored to the specific needs of affected communities.

## 5 Conclusion

This study provides valuable information about public health in Ecuador and underscores the importance of considering socioeconomic variables when addressing intestinal infectious diseases. Understanding these correlations and trends at the geographical level can contribute to improving health policies and medical care in the country.

In the future, more specific research should be conducted to identify the underlying causes of variability in the morbidity of these diseases, as well as more effective prevention strategies. Additionally, considering the correlations found, intervention programs could be designed to address socioeconomic variables to reduce the incidence of these diseases in specific areas. For instance, enhancing access to safe drinking water, adequate sanitation, and garbage collection services in areas with a high incidence of morbidity can significantly reduce the prevalence of these diseases. Given the observed spatial autocorrelation, it would be beneficial to consider implementing targeted and prioritized interventions in specific geographic areas. Furthermore, future studies should evaluate the impact of these strategies to ensure their effectiveness and suitability to the needs of the population.

## Data availability statement

Original datasets are available in a publicly accessible repository: The original contributions presented in the study are publicly available. This data can be found at the following links: https://www.ecuadorencifras.gob.ec/camas-y-egresos-hospitalarios-informacion-historica/ (20); https://www.ecuadorencifras.gob.ec/proyecciones-poblacionales/ (17); https://www.ecuadorencifras.gob.ec/censo-de-poblacion-y-vivienda/ (22); https://www.ecuadorencifras.gob.ec/nacimientos-y-defunciones-informacion-historica/ (23); https://educacion.gob.ec/base-de-datos/ (25); https://www.ecuadorencifras.gob.ec/nacimientos-y-defunciones-informacion-historica/ (26).

## Ethics statement

Ethical review and approval was not required for the study on human participants in accordance with the local legislation and institutional requirements. Written informed consent from the [patients/ participants OR patients/participants legal guardian/next of kin] was not required to participate in this study in accordance with the national legislation and the institutional requirements.

## Author contributions

KL: Conceptualization, Data curation, Formal analysis, Investigation, Methodology, Writing – original draft, Writing – review & editing. MC-M: Data curation, Formal analysis, Writing – original draft, Writing – review & editing. MS-M: Writing – original draft, Writing – review & editing. AC-A: Writing – review & editing. MD-C: Writing – review & editing. EQ: Conceptualization, Writing – review & editing.
